# Supplementary material for: Mutations in PCYT2 disrupt etherlipid biosynthesis and cause a complex hereditary spastic paraplegia
Source: Brain. 2019 Oct 22;142(11):3382–97. doi: 10.1093/brain/awz291 (PMC6821184; doi:10.1093/brain/awz291)
Supplement: awz291_Supplementary_Data [file awz291_supplementary_data.zip › awz291-Suppl_data/Supplementary_Data4.zip!/Supplementary information 4 - plasma lipidomics.zip!/Plasma lipidomics/Multiple comparison/PLS-DA_scores.pdf]

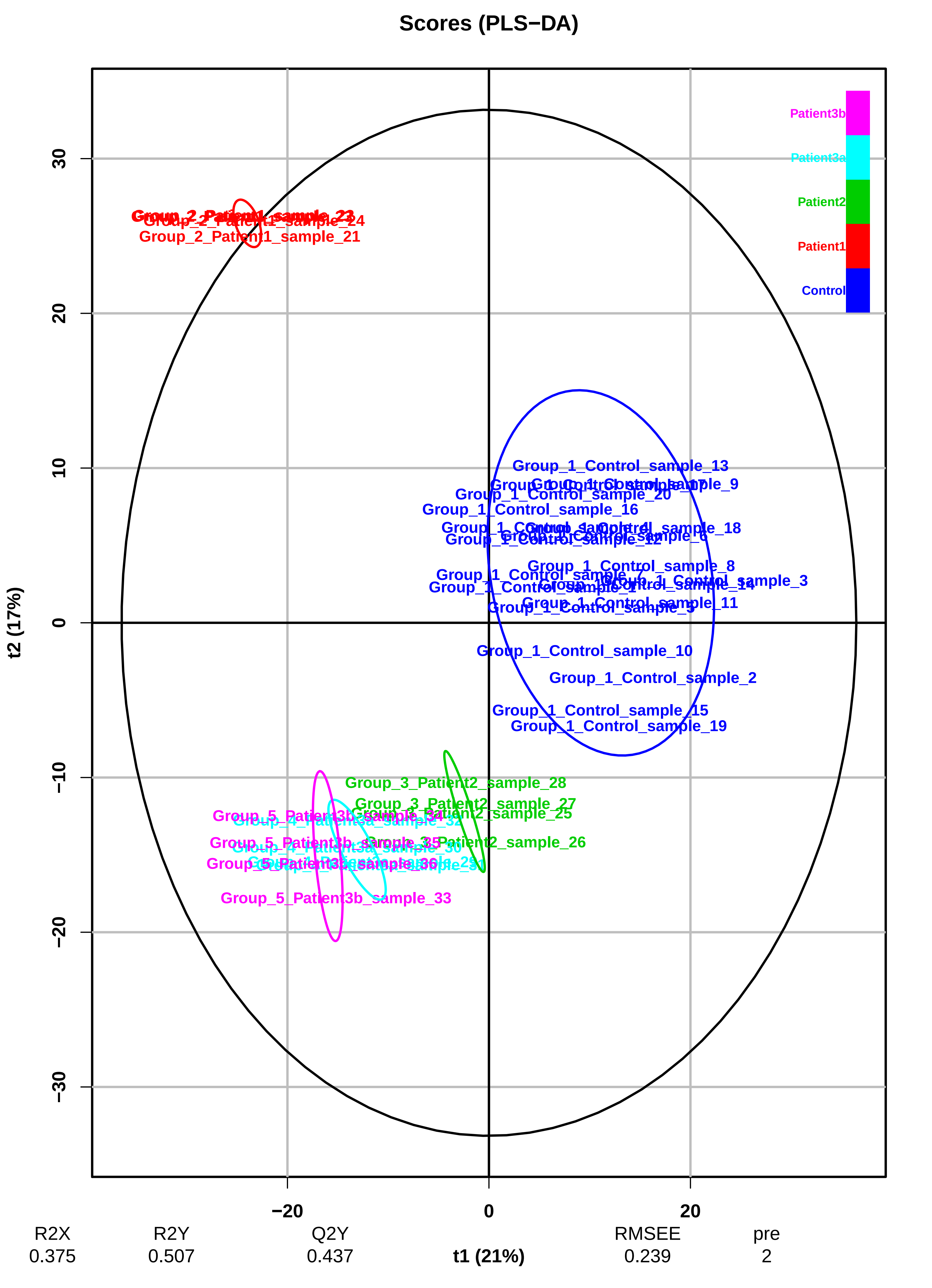

Scores (PLS−DA)
Patient3b
Patient3a
Patient2
Patient1
Control
Group_2_Patient1_sample_21
Group_1_Control_sample_13
Group_1_Control_sample_20
Group_1_Control_sample_16
Group_1_Control_sample_18
Group_1_Control_sample_8
Group_1_Control_sample_3
Group_1_Control_sample_11
Group_1_Control_sample_10
Group_1_Control_sample_2
Group_1_Control_sample_15
Group_1_Control_sample_19
Group_3_Patient2_sample_28
Group_3_Patient2_sample_27
Group_3_Patient2_sample_25
Group_3_Patient2_sample_26
Group_5_Patient3b_sample_33
t2 (17%)
t1 (21%)
30
20
10
0
−10
−20
−30
R2X
0.375
R2Y
0.507
Q2Y
0.437
RMSEE
0.239
pre
2
